# Pandemic Agreement Must Include Levers to Redirect Pharmaceutical Industry Behaviour During Pandemics: Comment on "More Pain, More Gain! The Delivery of COVID-19 Vaccines and the Pharmaceutical Industry’s Role in Widening the Access Gap"

**DOI:** 10.34172/ijhpm.8589

**Published:** 2024-09-09

**Authors:** Deborah Gleeson, James Scheibner, Brigitte Frances Tenni, Belinda Townsend, Dianne Nicol

**Affiliations:** ^1^Department of Public Health, School of Psychology and Public Health, La Trobe University, Melbourne, VIC, Australia.; ^2^Centre for Social Impact, College of Business Government and Law, Flinders University, Adelaide, SA, Australia.; ^3^Nossal Institute for Global Health, Melbourne School of Population and Global Health, University of Melbourne, Melbourne, VIC, Australia.; ^4^School of Psychology and Public Health, La Trobe University, Melbourne, VIC, Australia.; ^5^School of Regulation and Global Governance, Australian National University, Canberra, ACT, Australia.; ^6^Centre for Law and Genetics, Faculty of Law, College of Arts, Law and Education, University of Tasmania, Hobart, TAS, Australia.

**Keywords:** COVID-19 Vaccines, Pharmaceutical Industry, Pandemic Agreement, TRIPS Waiver, Intellectual Property, Pandemic-Related Products

## Abstract

Borges and colleagues rightly argue that an international treaty is needed to curtail the profit-driven behaviour of the pharmaceutical industry during pandemics. The Pandemic Agreement currently being negotiated by Member States of the World Health Organization (WHO) offers an important opportunity to equip nation states with greater leverage over industry behaviour. In this commentary, we examine the potential of current draft textual proposals for the Pandemic Agreement to redirect pharmaceutical behaviour in future pandemics. However, the future of the Agreement negotiations remains uncertain in the wake of the failure to conclude negotiations in time for the 2024 World Health Assembly (WHA). Further, there is limited consensus over proposals that could enable nation states to have greater leverage over industry behaviour. A concerted effort will need to be made to achieve a consensus text that shifts the status quo by giving nation states more power to curtail the self-interest of the pharmaceutical industry.

 Borges and colleagues^1^ highlight the role of the multinational pharmaceutical industry in the “grossly unequal” distribution of COVID-19 vaccines by documenting how its profit-driven behaviour undermined the supply of vaccines to COVAX and channelled doses overwhelmingly towards high-income countries (HICs). Their paper concludes with a call for an international treaty to regulate pharmaceutical industry activity and hold companies and other actors accountable for providing access to life-saving products during pandemics.

 As the global supply of COVID-19 vaccines increased over time, vaccination rates slowly increased in low-income countries. But inequities in access to COVID-19 response products remain a significant ongoing problem. By March 2024, four years into the pandemic, the proportion of people in low-income countries who had received one or more COVID-19 doses was still lagging way behind at 32.7%, in comparison with 70.6% of the global population and almost 80% of those in HICs.^2^ There also remains significant unmet need for COVID-19 therapeutics and diagnostic tests. In 2022, unmet need for the oral antiviral Paxlovid in low- and middle-income countries (LMICs) was estimated at 8 million courses, or 90% of the health need for this drug.^3^

 Almost two years on from the publication of Borges and colleagues’ analysis, the predictable effects of these inequities are now becoming clear. A retrospective modelling study showed that inequitable distribution of COVID-19 vaccines resulted in 1.3 million deaths in LMICs in 2021 alone.^4^ A further study of the impact of COVID-19 vaccine inequity in twenty LMICs found that over 50% of deaths due to COVID-19 in these LMICs would not have occurred if they had been able to achieve per capita vaccination rates equivalent to HICs.^5^ The health consequences of inequitable access to COVID-19 products also extend beyond the burden of disease and death from COVID-19 specifically to include many indirect health effects. These include excess mortality rates, diversion of health resources from other pressing health needs, and other health impacts arising from exacerbation of existing economic inequality and poverty.^6^

 At this stage, the world seems no closer to addressing the systemic barriers to equitable access or preventing a repeat of these inequities in future pandemics. As Borges et al point out, 20 months of negotiations over a proposed “TRIPS waiver” – a temporary suspension of intellectual property (IP) rights enshrined in the World Trade Organization’s (WTO’s) Agreement on Trade-Related Aspects of Intellectual Property Rights (TRIPS) that apply to COVID-19 products and technologies – resulted in a narrow Ministerial Decision^7^ that was “an ineffective compromise to address the issues raised by the waiver proponents.”^1^ While India and South Africa had proposed a waiver of all IP rights relevant to all COVID-19 response products, the Ministerial Decision was limited to vaccine patents and only provided a minor easing to TRIPS rules regarding the export of COVID-19 vaccines.^8^ Borges and colleagues’ warning has been borne out: in the two years since its adoption in June 2022, there have been no reports of its use. In February 2024, WTO Members acknowledged that consensus could not be reached on whether to expand the Decision to cover COVID-19 therapeutics and diagnostics, and brought a halt to further negotiations.^9^

## The Potential for the World Health Organization Pandemic Agreement to Address the Main Problematic Pharmaceutical Industry Behaviours During Pandemics

 The need for a robust international instrument that addresses the range of systemic barriers preventing equitable access to pandemic response products—as Borges et al argued so well in 2022—has never been clearer, and the process announced by Member States of the World Health Organization (WHO) in December 2021 to develop such a new legal instrument initially appeared very promising.^8^ Initial proposals for a pandemic treaty indicated that it would include a range of issues related to equitable access for pandemic-related products such as: research and development (R&D); transfer of technology and know-how; sustainable production; supply chains and logistics; access and benefit sharing; regulatory strengthening; and liability risk management. However, seeking agreement on many of these issues has proved extremely challenging. The deadline Member States set for themselves to finalise the draft for adoption at the World Health Assembly (WHA) in May 2024 was missed, but countries have decided to continue negotiations for up to one more year.

 Building on the analysis provided by Borges et al, we examine the main problems with behaviour of the pharmaceutical industry during COVID-19 that underpinned inequitable access to pandemic response products and evaluate how successful the proposed WHO Pandemic Agreement would likely be in addressing these problems if the draft currently under consideration is adopted.

## Problem Behaviour 1: Insistence on Conditions That Hamper Equitable Access in Contractual Agreements With Public Funders

 In the early stages of the pandemic, an unprecedented amount of public funding was funnelled into private pharmaceutical industry COVID-19 response products through government and charitable sources, including via R&D funding agreements and advance purchase agreements (APAs).^10^ However, as Borges et al^1^ point out, the pharmaceutical companies retained control over “…who can produce vaccines, the volume of vaccine doses, the prices charged, and whom to sell to through their APAs” (p. 3111). Non-disclosure obligations insisted on by multinational pharmaceutical firms hampered public access to information about the terms of these funding contracts and enabled inequitable pricing between countries to go undiscovered for some time.^11^ It gradually came to light through various leaks and information disclosures that pharmaceutical companies had insisted on a range of other conditions that impeded access to COVID-19 vaccines. These included: exclusive ownership of IP rights, in some cases even when product development was abandoned; restrictions on redistribution of doses; restrictions on the geographical location of production; and problematic indemnity clauses.^11,12^

 To address this issue, the Proposal for the WHO Pandemic Agreement released April 22, 2024^13^ included the following provision (Article 9, para 4):

 “*Each Party shall ensure that government-funded research and development agreements for the development of pandemic-related health products include, as appropriate, provisions that promote timely and equitable access to such products and shall publish the relevant terms. Such provisions may include: ( i ) licensing and/or sublicensing, preferably on a non-exclusive basis; (ii) affordable pricing policies; (iii) technology transfer on mutually agreed terms; (iv) publication of relevant information on research inputs and outputs; and/or (v) adherence to product allocation frameworks adopted by WHO.”*

 While this provision would require Parties to publish the terms of government-funded R&D agreements, the language about the inclusion of provisions promoting access is weak and voluntary. “As appropriate” leaves room for Parties to opt out entirely, and the list of provisions which “may” be included is optional. The requirement to publish conditions promoting access may not count for much if there is no corresponding requirement to include such provisions in contracts. The requirement to support the transparent and public sharing of research inputs and outputs from government funded research into pandemic-related products, as well as equitable access to knowledge, have also been removed. Removing these provisions undermines attempts to facilitate collaborative research into pandemic response products between LMICs and HICs. However, even this relatively weak provision has so far proved a sticking point in the negotiations. In the draft reflecting progress just before the talks were postponed at the WHA in May 2024,^14^ no convergence was reached on the paragraph (now Article 9, para 5) and it remained heavily bracketed. This bracketing indicates intense debate continues over the language of this paragraph.

## Problem Behaviour 2: The Industry’s Failure to Share IP, Technology and Know-How With Capable Manufacturers in LMICs

 As Borges et al^1^ and others have shown, the pharmaceutical industry eschewed participation in the COVID-19 Technology Access Pool established by the WHO during the emergency phase of the pandemic, and with a few notable exceptions, engaged in very limited initiatives to voluntarily share their IP, technology and know-how. Major mRNA vaccine manufacturers also declined to cooperate with the WHO mRNA Technology Transfer Hub established in South Africa.^6^

 Article 11 of the proposed Pandemic Agreement addresses technology transfer. However, it is also couched in optional, “best endeavours” terms. Although Article 11 para 1(f) addresses information for manufacturing pandemic response products, it only requires Parties to “urge” manufacturers to share this information in accordance with national laws and policies. Further, whether this sharing should be limited to circumstances where the lack of such information prevents or hinders those parties is contested.

 The May 2024 draft moved away from language in earlier drafts committing the Parties to “take appropriate measures to support time-bound waivers of IP rights” toward less specific text on supporting “time-bound measures to accelerate or scale up the manufacturing of pandemic-related health products” (Article 11, para 3). Likewise, proposals for requiring Parties to implement TRIPS flexibilities in their national laws (including research exemptions) and to ensure bilateral and regional trade agreements do not interfere with TRIPS flexibilities had been deleted. The flexibilities contained within the TRIPS Agreement, including compulsory licensing, are designed to mitigate the negative impacts of patents, such as high medicines prices caused by monopolies on new medicines. There are limitations to the utility of compulsory licensing during a pandemic. For example, compulsory licensing mechanisms only apply to patents and not manufacturing know-how, and separate licenses would need to be issued for each patent in each country.^15^ Nevertheless, the use of TRIPS flexibilities remains an important legal mechanism available to WTO Member States to protect public health^15^ especially in the absence of broader time-bound IP waivers.

 On TRIPS flexibilities, the only text remaining in the draft for which there was convergence by May 2024 is Article 11 para 4,^14^ stating:

 “*The Parties that are World Trade Organization (WTO) members reaffirm that they have the right to use, to the full, the TRIPS Agreement and the Doha Declaration on the TRIPS Agreement and Public Health of 2001, which provide flexibility to protect public health in future pandemics. The Parties respect the use of the TRIPS flexibilities that are consistent with the TRIPS Agreement.”*

 However, additional proposed wording in para 4 prohibiting Parties from exercising pressure to discourage the use of TRIPS flexibilities was still marked as text where the views of Parties diverged. This requirement for Parties to respect each other’s use of TRIPS flexibilities is important given the pressure countries have been put under not to use them in the past.^15^ Without it, the provision merely reasserts the rights Parties already have under the TRIPS Agreement, rather than providing new levers to ensure sharing of technology and know-how.

## Problem Behaviour 3: Prioritisation of Product Sales to Countries Able to Pay Market Prices

 As Borges and colleagues^1^ demonstrate so clearly, COVID-19 vaccine manufacturers prioritised sales to HICs, starving COVAX of doses. To address this issue, a Pathogen Access and Benefit Sharing system is included in the proposed Pandemic Agreement. In exchange for access to genetic information about pathogens, this system would channel a minimum of 20% of pandemic response products to WHO for distribution to LMICs (10% to be donated and 10% to be provided to WHO at affordable prices). However, even this modest proposal – which would not go very far in meeting access gaps like those seen during COVID-19 – has met with strenuous opposition from industry and some WHO Members, with Article 12 of the May 2024 draft showing little consensus on the “benefit” side of the Access and Benefit Sharing bargain. Further, the operation of the Pathogen Access and Benefit Sharing system would be subject to a future instrument that would still need to be negotiated.

## Conclusion

 As we have shown above, the various drafts of the Pandemic Agreement, while limited in some respects, have attempted to provide WHO Members with levers to address some of the most problematic pharmaceutical behaviours seen during COVID-19. These behaviours include conditions in contracts between public funders and private industry that hamper access to pandemic-related products, industry’s failure to share IP, technology and know-how, and prioritisation of sales to HICs. To solve these problems, nation states will need to push on towards a consensus text that shifts the status quo in these areas. This will require significant strengthening of the May 2024 draft.

## Ethical issues

 Not applicable.

## Conflicts of interest

 Authors declare that they have no conflicts of interest.
